# Supplementary material for: Organic Compounds as Corrosion Inhibitors for Carbon Steel in HCl Solution: A Comprehensive Review
Source: Materials (Basel). 2022 Mar 9;15(6):2023. doi: 10.3390/ma15062023 (PMC8954067; doi:10.3390/ma15062023)
Supplement: Supplementary file 1 [file materials-15-02023-s001.zip › materials-1593731-supplementary.pdf]

nanomaterials

MDPI

Table S1 Physicochemical and morphology characterization techniques for corrosion inhibitor investigations.

	Techniques	Abbreviations
1	Fourier transform infrared spectroscopy	FTIR
2	UV–visible spectroscopy	UV
3	Raman spectroscopy	Ram
4	X-ray diffraction spectroscopy	XRD
5	X-ray photoelectron spectroscopy	XPS
6	Scanning electron microscope	SEM
7	Atomic force microscopy	AFM
8	Transmission electron microscopy	TEM
9	Energy dispersive spectrometer	EDS
10	Digital light scattering	DLS
11	Laser scanning confocal microscopy	LSCM
12	Scanning probe microscopy	SPM
13	Optical microscopy	OM
14	Scanning kelvin probe	SKP
15	Thermogravimetric Analysis	TGA
16	Contact angle	CA
17	Ion chromatography	IC
18	Proton nuclear magnetic resonance spectroscopy	^{1}H NMR
19	Carbon nuclear magnetic resonance spectroscopy	^{13}C NMR
20	Fluorine nuclear magnetic resonance spectroscopy	^{19}F NMR
21	Distortionless enhancement by polarization transfer	DEPT NMR
22	Mass spectrum	MS
23	High resolution mass spectrum	HRMS
24	Gas chromatography coupled with mass spectrometry	GC–MS
25	Liquid chromatography coupled with mass spectrometry	LC–MS
26	Centrifugal partition chromatography	CPC
27	High-performance liquid chromatography	HPLC
28	Thin-layer chromatography	TLC
29	Rheological mechanical spectrometer	RMS
30	Gel permeation chromatography	GPC
31	Zeta potential analysis	-
32	Capillary viscometry	-
33	Surface tension	-
34	Emulsification power	-
35	Foam power	-
36	Conductivity method	-
37	Ubbelhode capillary viscometer	-
38	Biodegradability test	-
39	Biocorrosion monitoring	-
40	Antimicrobial assay	-

Table S2. Drugs as corrosion inhibitors for carbon steel in 1.0 M HCl media, the techniques, methods and instruments used for evaluation of the inhibition performance, and the results obtained.

Inhibitor name	Sample	Optimum concentration	Techniques/ Investigations	Max Efficiency (%)	T (°C)	Electrochemical Type	Sorption	Isotherm model	Ref

1	(Z)-4-(4-hydroxy-3-methoxybenzylidene)-3-methylisoxazol-5(4H)-one	Mild steel	300 ppm	^{1}H NMR; WL; EIS; PDP; FTIR; AFM; SEM; EDS	96.6	30	Mixed type of inhibitor	Chemical	Langmuir	258
2	L-cysteine	Mild steel	5 mM	EIS; PDP; SEM; DFT; MD	91	-	Mixed type of inhibitor	Physical–chemical	Langmuir	142
3	Penicillin G	Carbon steel	10 mM	EDX; EN; EIS; PDP; SEM; FTIR	98.4	25	Mixed type of inhibitor	Physical	Langmuir	139
4	Fluconazole	X52 steel	20 ppm/200 ppm	EIS; PDP; AFM; SEM; XPS; DFT; MEP	89.9/90.6	20	Mixed type of inhibitor	Physical–chemical	Langmuir	146
5	Analgin	Mild steel	4000 ppm	PDP; EIS; WL; MD; SEM; AFM	96.1	25	Mixed type of inhibitor	Physical	Langmuir	165
6	Pheniramine	Mild steel	0.833 mM	WL; PDP; EIS; SEM; DFT	98.1	35	Mixed type of inhibitor	Physical–chemical	Langmuir	149
7	Phenylephrine	Mild steel	4000 ppm	PDP; EIS; WL; DFT; MC; AFM	88	25	Mixed type of inhibitor	-	Langmuir	259
8	Phenobarbital [5-ethyl-5-phenylpyrimidine-2,4,6(1H,3H,5H)-trione]	Mild steel	200 ppm	WL; DFT	95	35	-	Chemical	Langmuir	260
9	4-chloro-1H-pyrazolo[3,4-d]pyrimidine	Mild steel	1×10^{-3} M	PDP; EIS; OM; DFT	92.7	30	Mixed type of inhibitor	Chemical	Langmuir	261
10	Acrylamide methyl ether	Mild steel	500 mg/L	WL; PDP; EIS; DFT	98	25	Mixed type of inhibitor	Chemical	Langmuir	262
11	Expired Ambroxol drug	Mild steel	9 v/v%	WL; PDP; EIS; SEM; EDX; FTIR; DFT	94.03	-	Mixed type of inhibitor	Chemical	Temkin and Langmuir	254
12	Amlodipine Besylate	Carbon steel	250 ppm	WL; PDP; EIS; EFM; SEM; EDX; QM	84	30	Mixed type of inhibitor	Physical	Langmuir	263
13	(3R,5R)-7-(2-(4-fluorophenyl)-5-isopropyl-3-phenyl-4-(phenylcarbamoyl)-1H-pyrrol-1-yl)-3,5-dihydroxyheptanoic acid (Atorvastatin)	Mild steel	150 ppm	PDP; EIS; WL; SEM	99.08	-	Mixed type of inhibitor	Physical–chemical	Langmuir	264
14	((±)-[3-(9H-carbazol-4-yloxy)-2-hydroxypropyl][2-(2-methoxyphenoxy)ethyl]amine) (Carvedilol drug)	Carbon steel	1.6×10^{-4} M	WL; PDP; EIS; EFM; AFM	98.9	25	Mixed type of inhibitor	Physical–chemical	Langmuir	265
15	Expired asthalin drug	Mild steel	9 v/v%	WL; PDP; EIS; SEM	94.76	-	Mixed type of inhibitor	-	Langmuir	266
16	Semisynthetic antibiotic cloxacillin	Mild steel	15×10^{-4} M	WL; PDP; EIS; HE; UV	81.19	-	Mixed type of inhibitor	-	Temkin	267
17	Streptomycin	Mild steel	500 ppm	WL; PDP; EIS; AFM	88.5	35	Mixed type of inhibitor	-	Langmuir	152
18	Atenolol ((RS)-2-{4-[2-Hydroxy-3-(propan-2-ylamino)propoxy]phenyl}acetamide)	Mild steel	300 ppm	EIS; PDP; WL; QM; SEM; FTIR	93.8	-	Mixed type of inhibitor	Physical	Langmuir	156

19	Expired Tramadol	Mild steel	100 mg·L^{-1}	W; PDP; EIS; AFM; SEM; DFT; MEP	97.2	35	Mixed type of inhibitor	Physical–chemical	Langmuir	158
20	Sulfa drugs (sulfadiazine)	Mild steel	5 mM	WL; PDP	94	25	Mixed type of inhibitor with a predominant cathodic	-	-	160
21	Cefalexin	Mild steel	400 ppm	WL; PDP; EIS; AFM	92.1	35	Mixed type of inhibitor	Physical	Langmuir	164
22	Losartan potassium	Q235 steel	5 mM	OCP; PDP; EIS; SVET; W; SEM; AFM; XPS; DFT; MD; RDF	94	45	Mixed type of inhibitor	Physical–chemical	Langmuir	78
23	Piroxicam	Mild steel	600 ppm	WL; DFT; SEM	86.9	25	Mixed type of inhibitor	Physical–chemical	Langmuir	166
24	Ceftriaxone	Mild steel	400 ppm	WL; PDP; EIS	92.59	-	Mixed type of inhibitor	Physical	Langmuir	171
25	Irbesartan drug	Mild steel	300 mg·L^{-1}	OCP; PDP; EIS; CV; UV; FTIR; ^{1}H NMR; SEM	95	-	Mixed type of inhibitor	Physical–chemical	Langmuir	70
26	Venlafaxine	Mild steel	4000 ppm	WL; EIS; PDP; MD; DFT; AFM	86.8	25	Mixed type of inhibitor	Physical	Langmuir	168
27	Fexofenadine (4-{1-hydroxy-4-(4-(hydroxy-diphenyl-methyl)-1-piperidyl)bu-tyl}phenyl]-2-methyl-propanoic acid)	Mild steel	3.0 × 10^{-4} M	EIS; PDP; WL; FTIR; DFT	98	-	Mixed type of inhibitor	Chemical	Langmuir	151
28	Pioglitazone drug	Mild steel	14 × 10^{-4} M	WL; PDP; EIS; SEM	79.51	30	Mixed type of inhibitor	-	-	169
29	Clozapine	Mild steel	1 × 10^{-3} M	PDP; EIS; WL; DFT; MD; RDF; SEM	97.46	30	Mixed type of inhibitor	Physical–chemical	Langmuir	268
30	Non-toxic bisacodyl (Bis (p-acetoxyphenyl)-2-pyridylmethane (Bisacodyl))	Mild steel	300 ppm	WL; OCP; PDP; EIS; SEM; EDX; AFM; FTIR; DFT; MD	92.62	30	Mixed type of inhibitor	Physical	Langmuir and Temkin	81
31	Moxifloxacin antibiotic	Carbon steel	300 ppm	WL; HE; PDP; EIS; EFM; SEM; EDX;	94.1	25	Mixed type of inhibitor	Physical	Langmuir	67
32	Dapsone's Schiff's base with salicylaldehyde (1:2)	Mild steel	500 ppm	WL; EIS; PDP; QM	97	-	Mixed type of inhibitor	Physical	Langmuir	269
33	Benzohydrazide Schiff bases ((Z)-2-hydroxy-N′-(2-oxo-1,2-diphenylethylidene) benzohydrazide)	Carbon steel	300 ppm	WL; PDP; EIS; SEM; EDX; DFT; MD	96.5	25	Mixed type of inhibitor	Physical–chemical	Langmuir	270
34	Quercetin	Mild steel	800 ppm	FTIR; UV; SEM; AFM; OCP; PDP; EIS; WL; MC; MD; DFT	95	25	Mixed type of inhibitor	Physical–chemical	Langmuir	271
35	Schiff base ((E,E)-N,N′-dibenzo[b,d]thiene-2,8-	Mild steel	1 × 10^{-3} M	FTIR; ^{1}H NMR; ^{13}C NMR; WL;	92.95	25	Mixed type of inhibitor	Physical–chemical	Langmuir	272

	diylbis[1-(thiophen-2-yl)methanimine])			PDP; EIS; SEM; DFT						
36	Carboxamide ligands ((N-(quinolin-8-yl) quinoline-2-carboxamide))	Mild steel	100 ppm	OCP; EIS; FTIR; SEM	94.34	25	-	Physical–chemical	Langmuir	273
37	Omeprazole	C38 Steel	5 × 10^{-3} M	WL; PDP; EIS; DFT; MD; RDF; SEM	98.36	25	Mixed type of inhibitor	Chemical	Langmuir	170
38	Expired anti-tuberculosis drug (Schiff bases of Isoniazid with 5-substitute indole derivatives)	Mild steel	200 mg·L^{-1}	FTIR; ^{1}H NMR; WL; EIS; PDP; SEM; EDX; AFM; XPS; DFT; MD	99.3	-	Mixed type of inhibitor	Physical–chemical	-	274
39	Niclosamide drug (5-chloro-N-(2-chloro-4-nitrophenyl)-2-hydroxyenzamide)	C-steel	50 µM	WL;PDP; EIS; EFM; SEM; EDX; AFM; FTIR; QM; MC	99.0	30	Mixed type of inhibitor	Physical–chemical	Langmuir	275
40	Antibiotic drug - ampicillin ((2S,6R)-6-(2-(aminomethyl)benzamido)-3,3-dimethyl-7-oxo-4-thia-1-azabicyclo(3.2.0) heptanes-2-carboxylic acid)	Mild steel	5 × 10^{-3} M	WL; QM	75.85	30	-	Physical	Langmuir	276
41	Expired pharmaceutical gentamicin drug	Mild steel	0.9 v/v%	WL; PDP; EIS; SEM	92.36	30	Mixed type of inhibitor	Chemical	Langmuir	277
42	Non-steroidal anti-Inflamma-tory drug - Piroxicam	Mild steel	600 ppm	EIS; PDP; QM; SEM	91.98	25	Mixed type of inhibitor	Physical–chemical	Langmuir	278
43	Modazar drug: Hydrochloro-thiazide (25 mg)/Losartan (100 mg)	Carbon steel	300 ppm	OCP; WL; HE; PDP; EIS; EFM; EDX; SEM; AFM	92.3	25	Mixed type of inhibitor	Physical	Langmuir	279
44	Torsemide drug	Mild steel	14 × 10^{-4} M	WL; PDP; EIS; EDX; SEM; AFM	91.68	-	Mixed type of inhibitor	Physical–chemical	Langmuir	280
45	Expired indocin-SR drug	Carbon steel	500 ppm	WL; OCP; PDP; EIS; SEM	83.81	25	Mixed type of inhibitor	Physical–chemical	Langmuir	281
46	Ketoconazole drug (1-[4-(4-{[(2R,4S)-2-(2,4-Dichloro-phenyl)-2-(1H-imidazol-1-ylmethyl)-1,3-dioxolan-4-yl]methoxy}phenyl)piperazin-1-yl]ethan-1-one)	Mild steel	100 ppm	WL; EIS; PDP; SKP; SEM; AFM; QM	94	-	Mixed type of inhibitor	-	Langmuir	282
47	Megavit zinc drugs	C-steel	300 ppm	COP; PDP; EIS	91.7	30	Mixed type mainly cathodic	-	Langmuir	283

Table S3. Ionic liquids as corrosion inhibitors for carbon steel in 1.0 M HCl media, the techniques, methods and instruments used for evaluation of the inhibition performance, and the results obtained.

	Inhibitor name	Sample	Optimum concentration	Techniques/ Investigations	Max Efficiency (%)	T (°C)	Electrochemical Type	Sorption	Isotherm model	Ref
1	1-butyl-4-(2-(4-fluorobenzylidene)hydrazinecarbonyl)pyridin-1-ium iodide	Mild steel	10^{-3} M	PDP; EIS; DFT; MD; RDF	92.30	25	Anodic type inhibitors	Chemical	Langmuir	284

2	3-(4-chlorobenzoylmethyl)-1-methylbenzimidazoliumbromide	Carbon steel	6.84 × 10^{-4} M	FTIR; ^{1}H NMR; ^{13}C NMR; EIS; PDP; EN; HE; SEM; EDX; DFT	97.9	–	Mixed type of inhibitor	Physical–chemical	Langmuir	285
3	1-butyl-1H-imidazol-1-ium 3-carboxy-2-((2-carboxylatoethyl)thio)propanoate	A3 steel	10 mM	TGA; WL; FTIR; XPS; SEM; DFT; CA; QM	90.11	25	-	Physical–chemical	Langmuir	286
4	Benzyltributylammonium tetrachloroferrate	Carbon steel	400 ppm	EIS; PDP; SEM; FTIR; UV; SEM; HE; DFT; MC	99.50	-	Mixed type of inhibitor	-	Langmuir	183
5	3-Dodecyl-1,2-dimethyl-1H-imidazol-3-ium tetrafluoroborate	Carbon steel	200 ppm	PDP; EIS; FTIR; XRD; ^{1}H NMR; TGA	92.8	25	Anodic type inhibitors	Physical	Temkin	287
6	5-(Trifluoromethyl)dibenzothiopheniumtrifluoromethanesulfonate	Mild steel	8.0 × 10^{-2} M	PDP; EIS; FTIR; SEM; EDS; WL; DFT; QM; MC	90.2	30	Mixed type of inhibitor (mainly anodic)	Physical	Langmuir	288
7	2-hydroxyethyl-trimethyl-ammonium acetate	Mild steel	17.91 × 10^{-4} M	WL; PDP; EIS; SEM; AFM; EDX; DFT; MC	96.59	-	Mixed type of inhibitor	Physical–chemical	Temkin	41
8	Tetra-n-butyl ammonium methioninate	Mild steel	1.59 × 10^{-3} M	PDP; EIS;EN; DFT; QM; SEM; EDX	95.1	-	Mixed type of inhibitor	Physical	Frundlich	64
9	(E)-4-(2-(4-fluorobenzylidene)hydrazinecarbonyl)-1-propylpyridin-1-ium iodide	Mild steel	10^{-3} M	EIS; PDP; DFT; MD; RDF; SEM	90.1	25	Anodic type inhibitors	Physical–chemical	Langmuir	289
10	1-ethyl-3-methylimidazolium ethylsulfate	Mild steel	500 ppm	PDP; EIS; UV; FTIR; SEM; QM; QSAR; MC	92.76	-	Mixed type of inhibitor (mainly cathodic)	Physical–chemical	Langmuir	290
11	1-(6-ethoxy-6-oxohexyl)pyridazin-1-ium bromide	Mild steel	10^{-3} M	EIS; MD	84	30	-	Chemical	Langmuir	291
12	3-(4-chlorophenylacylmethyl)-1-ethylbenzimidazolium tetrafluroborate	Carbon steel	250 ppm	^{1}H NMR; FTIR; EN; HE; SEM	97.4	30	-	Physical–chemical	Langmuir	292
13	1-butyl-3-methylimidazolium bromide	Mild steel	20 mM	WL; PDP; EIS	94	25	Mixed type of inhibitor	Physical	Langmuir	293
14	3-hexadecyl-1,2-dimethyl-1H-imidazol-3-ium bromide	Mild steel	250 ppm	^{1}H NMR; FTIR; WL; PDP; EIS; UV; SEM; EDX; AFM	87.12	25	Mixed type of inhibitor	Physical–chemical	Langmuir	294
15	1-methyl-3-(2-{2-[2-(1-methyl-1-H-imidazol-3-ium-3-yl)ethoxy]ethoxy}-ethyl-1H-imidazol-3-ium dichloride	Mild steel	200 mg·L^{-1}	WL; PDP; EIS; Surface tension measurements; AFM; SEM; CA	77	-	Mixed type of inhibitor (mainly anodic)	Physical–chemical	Langmuir	295
16	1-butyl-3-methylimidazolium hydrogen sulfate	Mild steel	1 × 10^{-2} M	^{1}H NMR; WL; EIS; PDP	93.6	30	Mixed type of inhibitor	Physical	Langmuir	296

17	1-Hexyl-3-methylimidazolium iodide	Mild steel	5×10^{-3} M	EIS; PDP; EFM; SEM; AFM; FTIR; QM	93.6	25	Mixed type of inhibitor	Physical	Langmuir	297
18	1-ethyl-3-phenethylimidazol-3-ium bromide	Carbon steel	5×10^{-3} M	^{1}H NMR; ^{13}C NMR; LC–MS; WL; PDP; SEM	80.9	25	Mixed type of inhibitor	Physical	Langmuir	298
19	1-hydroxyethyl-3-2011methylimidazolium hexafluorophosphate	Mild steel	16 mM	Conductivity method; EIS; PDP; SEM; UV; MD	79.94	25	Mixed type of inhibitor (mainly cathodic)	Physical	Langmuir	299
20	1-decyl-3-methylimidazolium tetrafluoroborate	Mild steel	500 ppm	PDP; EIS; UV; FTIR; Ram; SEM; QM; MC	98.08	30	Mixed type of inhibitor	Physical	Langmuir	177
21	1-butyl-3-methylimidazolium acetate	Mild steel	8.67×10^{-4} M	FTIR; ^{1}H NMR; ^{13}C NMR; WL; OCP; PDP; EIS; AFM; SEM; DFT; MD	97.15	-	Mixed type of inhibitor (mainly anodic)	-	Temkin	300
22	1-vinyl-3-aminopropylimidazolium hexafluorophosphate	Q235 Carbon steel	0.8 mM	^{1}H NMR; ^{13}C NMR; FTIR; Surface tension measurements; Conductivity method; WL; EIS; PDP; SEM; EDX; CA; UV; MD	90.53	45	Mixed type of inhibitor (mainly cathodic)	Physical–chemical	Langmuir and EI-Awady kinetic-therodynamic	174
23	1-Methyl-1-octyl-pyrrolidinium dicyanamide	Mild steel	5×10^{-3} M	PDP; EIS; SEM; FTIR; QM	96.21	25	Mixed (mainly cathodic)	Physical–chemical	Langmuir	178
24	3-((4-amino-2-methylpyrimidin-5-yl)methyl)-5-(2-hydroxyethyl)-4-methylthiazol-3-ium chloride	Carbon steel	40 ppm	WL; XRD; QM; SEM; EDS; OCP	91.40	-	Mixed type of inhibitor	Chemical	Langmuir	185
25	1-hexylpyridinium bromide	Carbon steel	3×10^{-3} M	WL; LPR; EIS; SEM	88.6	21	Mixed type of inhibitor	Physical	Langmuir	180
26	1-(3-bromopropyl)-4-(dimethylamino)pyridinium bromide	Carbon steel	3×10^{-3} M	^{1}H NMR; ^{13}C NMR; DEPT; WL; LPR; EIS	87.1	23	Mixed type of inhibitor	Physical	Langmuir	179
27	4-(dimethylamino)-1-(6-methoxy-6-oxohexyl)pyridinium bromid	Carbon steel	1×10^{-3} M	^{1}H NMR; ^{13}C NMR; FTIR; LC–MS; WL; EIS; PDP; DFT; SEM	93	30	Mixed type of inhibitor	Chemical	Langmuir	301
28	3-amino-7-(dimethylamino) phenothiazin-5-ium chloride	Mild steel	5×10^{-5} M	EIS; LPR; PDP; SEM; DFT	96.90	25	Mixed type of inhibitor	Chemical	Langmuir	186
29	1-(2-(4-chlorophenyl)-2-oxoethyl)pyridazinium bromide	Carbon steel	1×10^{-3} M	^{1}H NMR; ^{13}C NMR; FTIR; LC–MS; WL; EIS; PDP; QM; OM	91.67	25	Mixed type of inhibitor	Chemical	Langmuir	181
30	1-(3-phenoxypropyl) pyridazin-1-ium bromide	Mild steel	1×10^{-3} M	WL; EIS; SEM; XPS	91.03	25	-	-	-	302

31	1-(2-(4-nitrophenyl)-2-oxoethyl) pyridazinium bromide	Carbon steel	1×10^{-3} M	WL; PDP; EIS; OM	88	45	Mixed type of inhibitor	Chemical	Langmuir	303
32	Poly [3-butyl-1-vinylimidazolium bromide]	Mild steel	40 ppm	OCP; EIS; PDP; SEM; DFT	96	-	Mixed type of inhibitor	Physical	Frumkin	304
33	Polymeric ionic liquids: CSPTA-lauric	Carbon steel	250 ppm	FTIR; ^{1}H NMR; HE; PDP; EIS; WL; SEM; EDX; DFT	98.7	-	Mixed type of inhibitor	-	-	193
34	Poly-2-acrylamido-2-methylpropane sulfonic acid triethanolamine derivative	Carbon steel	250 ppm	FTIR; ^{1}H NMR; ^{13}C NMR; HE; PDP; EIS; SEM	91.4	-	Mixed type of inhibitor	Physical–chemical	-	66
35	1-propyl-2,3-methylimidazolium bis(trifluoromethyl-sulfonyl) imide	Mild steel	500 ppm	WL; PDP; EIS; QM; QSAR; DFT	75.9	30	Mixed type of inhibitor	Physical–chemical	Langmuir	305
36	1-methyl-3-(2′-aminoethane)imidazolium bromide modified carbon dots (IM-CDs)	Q235 steel	200 mg·L^{-1}	FTIR; UV; Ram; XPS; TEM; SPM; OCP; EIS; PDP; SVET; SEM; EDS; LS-CM; WL	92.6	-	Cathodic type	Physical–chemical	Langmuir	79
37	1-hexyl-3-methylimidazolium trifluoromethanesulfonate	Mild steel	500 ppm	PDP; EIS; FTIR; UV; QM	81.16	30	Mixed type of inhibitor	Physical–chemical	Temkin	173
38	Methyltrioctylammonium methyl sulfate	X52 steel	100 ppm	^{1}H NMR; ^{13}C NMR; OCP; EIS; PDP; XPS; SEM; AFM	94 ± 2	30	Mixed type of inhibitor	Physical	Langmuir	306
39	L-Phenyl Alanine methyl ester saccharinate	Mild steel	100 ppm	WL; PDP; EIS; OCP; FTIR; CA; SEM; EDS; DFT; MC	93.2	60	Mixed type of inhibitor	Physical	Langmuir	307
40	1,3-dibencilimidazolio dodecanoate	API 5LX52 steel	100 ppm	PDP; EIS; SEM	88	-	Mixed type of inhibitor	Physical–chemical	Langmuir	14

Table S4. Surfactants as corrosion inhibitors for carbon steel in 1.0 M HCl media, the techniques, methods and instruments used for evaluation of the inhibition performance, and the results obtained.

	**Inhibitor name**	**Sample**	**Optimum concentration**	**Techniques/ Investigations**	**Max Efficiency (%)**	**T (°C)**	**Electrochemical Type**	**Sorption**	**Isotherm model**	**Ref**
1	Bis(p-(N,N,N-dodecyldimethylammonium bromide)benzylidene)benzene-1,4-diamine	Carbon steel	5×10^{-3} M	FTIR; ^{1}H NMR; EIS; PDP; WL; SEM	94.51	30	Mixed type of inhibitor	Physical–chemical	Langmuir	308
2	N,N′-((oxalylbis(oxy))bis(ethane-2,1-diyl))bis(N,N-dimethyldodecan-1-aminium bromide)	Carbon steel	5×10^{-3} M	FTIR; ^{1}H NMR; MS; WL; EIS; PDP; Surface tension measurement	94.84	25	Mixed type of inhibitor	Physical–chemical	Langmuir	89

3	(diethylhexanedioate)diyl-α,ω-bis(dimethyl myristyl ammonium bromide)	Carbon steel	5.0×10^{-4} M	Conductivity method; Surface tension measurements; ^{1}H NMR; WL	96.39	35	-	Chemical	Langmuir	309
4	N,N′-bis(2-hydroxyethyl)-N,N′-dimethyl-N,N′-bis (2-(tetradecanoyloxy) ethyl) dodecane-1,12-diaminium bromide	Carbon steel	5×10^{-3} M	PDP; EIS; Biodegradability test	≈99	25	Mixed type of inhibitor	Physical–chemical	Langmuir	310
5	N1,N1,N1,N2,N2,N2-hexadodecylhexane-1,6-diaminium bromide	Carbon steel	5×10^{-3} M	FTIR; ^{1}H NMR; Surface tension measurement; PDP; EIS; WL	93	25	Mixed type of inhibitor	Physical–chemical	Langmuir	311
6	N1,N1,N3,N3-tetramethyl-N1,N3-bis(2-(tetradecanoyloxy)ethyl)propane-1,3-diammonium bromide	Carbon steel	1×10^{-3} M	^{1}H NMR; FTIR; Ram; Surface tension measurements; Conductivity method; Zeta potential measurement; WL	99.1	25	-	-	-	312
7	Decamethylene bis-pyridinium dibromide	P110 steel	300 mg/L	PDP; EIS; WL; SEM; CA; DFT	91.76	30	Mixed type of inhibitor	Physical–chemical	Langmuir	313
8	N,N′-((ethane-1,2-diylbis(oxy))bis(2-oxoethane-2,1-diyl))bis(N,N-dimethyl-4-((E)-(2-((E)-octadec-9-enoyl)hydrazineylidene)methyl)benzenaminium) dichloride	X-65 steel	1×10^{-4} M	FTIR; ^{1}H NMR; ^{13}C NMR; Surface tension measurement; EIS; PDP; DFT; MC; SEM; AFM; XRD	95	25	Mixed type of inhibitor	Physical	Freundlich	82
9	Ethane-1,2-diyl bis(N,N-dimethyl-N-tetradecylammoniumacetoxy) dichloride	Mild steel	1×10^{-2} M	FTIR; ^{1}H NMR; Surface tension measurement; WL; PDP; EIS; UV; SEM; EDX; TGA; QM	96.79	30	Mixed type of inhibitor	Chemical	Langmuir	314
10	SH1500 (2-mercaptoacetic acid and polyethylene glycol-1500 were esterified individually in xylene)	Mild steel	1×10^{-2} M	FTIR; ^{1}H NMR; WL; PDP; EIS	79.29	25	Mixed type of inhibitor	Physical–chemical	Langmuir	315
11	Nonionic surfactant VI (Sebacic acid–antipyrine amide and polyethylene glycol with 2000 g/mol were reacted individually at equimolar ratios in 100 mL xylene as a solvent and p-toluene sulfonic acid (0.01 g) as a dehydrating agent)	Mild steel	800 ppm	FTIR; ^{1}H NMR; Surface tension measurement; WL	98.5	25	-	-	-	316
12	L-cysteine + Triton X-100 surfactant	Mild steel	500 ppm + 1 ppm	WL; PDP; EIS; FTIR; SEM	98.58	30	Mixed type of inhibitor	Physical	Langmuir	317
13	P-benzylidene benzyldodecyl iminium chloride	Carbon steel	5×10^{-4} M	^{1}H NMR; Conductivity	98.48	60	Mixed type of inhibitor	Chemical	Langmuir	318

				method; WL; UV; PDP; EIS; QM; SEM; EDX						
14	Ethane-1,2-diylbis(N,N-dimethyl-N-alkylammoniumacetoxy)dichloride (16-E2-16)	Mild steel	500 ppm	WL; PDP; EIS; FTIR; SEM; MD; RDF	91.61	30	Mixed type of inhibitor	Chemical	Langmuir	211
15	Chitosan-R16 surfactant (anionic polymeric surfactant based on ecofriendly chitosan materials)	Carbon steel	800 ppm	FITR; UV; Surface tension measurement; WL; PDP; EIS; AFM; XPS	92.62 ± 1.29	25	Mixed type of inhibitor	Physical–chemical	Langmuir	202
16	Nonionic surfactants S2 (phenyl alanine were reacted with oleic acid and polyethylene glycol (600))	Carbon steel	1×10^{-3} M	FITR; ^{1}H NMR; ^{13}C NMR; Surface tension measurement; PDP; EIS; QM	94.14	25	Mixed type of inhibitor	Physical	Langmuir	319
17	1,2-ethane bis(dimethyl alkyl ammonium bromide) (12-2-12 GS)+n-butanol (1:1)	Mild steel	1 mM (12-2-12 GS)+1 mM (C_4OH)	^{1}H NMR; WeL; PDP; EIS; FITR; AFM; SEM; EDX; QM	98.76	30	Mixed type of inhibitor	Chemical	Langmuir	320
18	Novel hybrid cationic surfactants F6H6 (synthesized using perfluorobutylsulfonyl or perfluorohexylsulfonyl and hexyl as the hybrid chains, connected via a cationic bridge of N-benzyl-N′,N′-dimethyl ethylenediamine)	Carbon steel	1.5×10^{-4} M	^{1}H NMR; ^{19}F-NMR; FTIR; HRMS; Surface tension measurement; PDP; EIS; SEM; XPS	95.80	25	Mixed type of inhibitor	Physical–chemical	Langmuir	321
19	1,12-bis((1H-benzimidazol-2-thioyl)dodecane	Mild steel	5×10^{-4} M	^{1}H NMR; ^{13}C NMR; FITR; WL; EIS; PDP; LPR; SEM; DFT; MD; RDF	97	30	Mixed type of inhibitor	Physical–chemical	Langmuir	322
20	N-(2-(3,4-dimethoxybenzylideneamino) ethyl)-N,N-dimethylhexadecan-1-ammonium bromide	Mild steel	5×10^{-3} M	WL; PDP; EIS; SEM; EDX; DFT	93.71	70	Mixed type of inhibitor	Chemical	Langmuir	323
21	Synthesized surfactant VSBH (C31H47O4N2Br) (3-aminopyridine–vanillin Schiff base and hexadecyl bromoacetate esters were refluxed individually in acetone (100 mL) as a solvent for 4 h and left to cool)	Carbon steel	400 ppm	FITR; ^{1}H NMR; ^{13}C NMR; Surface tension measurements; Emulsification power; Biodegradation test; WL; PDP	95	25	Mixed type of inhibitor	Physical	Langmuir	324
22	4,4′-((1E,1′E)-((3,3′-dimethoxy-[1,1′-biphenyl]-4,4′-diyl)bis(azaneylylidene))bis(methaneylylidene))bis(N-dodecyl-N,N-	Carbon steel	1×10^{-3} M	FITR; ^{1}H NMR; PDP; EIS; EFM	99.56	25	Mixed type of inhibitor	Physical	Langmuir	61

	dimethylbenzenaminium)bromide									
23	Bis(17-hydroxy-3,6,9,12,15-pentaoxaheptadecyl) 4,4′(3,3′-thiocarbonylbis(azan-1-yl-1-ylidene) bis (methan-1-yl-1-ylidene) bis (4-hydroxy-3,1-phenylene)) bis (diazene-2,1-diyl) dibenzenesulfonate	Carbon steel	7.5×10^{-4} M	FITR; ^{1}H NMR; Surface tension measurement; PDP; EIS; EFM; UV; SEM; QM; MD	94.11	25	Mixed type of inhibitor	Physical	Langmuir	199
24	N-(3-(dimethyl benzyl ammonio)propyl)palmitamide chloride	Carbon steel	5 × 10^{-4} M	FITR; ^{1}H NMR; WL; PDP; EIS; DFT	95.16	60	Mixed type of inhibitor	Chemical	Langmuir	325
25	(E)-N-(3-((4-hydroxy-3-methoxybenzylidene)amino)propyl)-N,N-dimethylhexadecan-1-aminium	Carbon steel	1 × 10^{-2} M	FTIR; ^{1}H NMR; MS; WL; PDP; EIS	96.18	70	Mixed type of inhibitor	Physical–chemical	Langmuir	326
26	Ethoxylated N-(2-(2-aminoethylamino)ethyl)octadec-9-enamide (ET-40)	API X65 steel	0.5 mM·dm^{-3}	FTIR; WL; PDP; EIS; Surface tension measurement; SEM; EDX	92.3	30	Mixed type of inhibitor	Physical	Langmuir	327
27	Alginates polymeric cationic surfactants (ALGHB)	Carbon steel	1000 ppm	FTIR; ^{1}H NMR; WL; Surface tension measurement; Foam power; Emulsification power; PDP; EIS; Biodegradability test	89.31	70	Mixed type of inhibitor	Physical–chemical	Langmuir	328
28	Fluorinated surfactants BFIS (hexafluoropropene trimer or perfluorohexanesulfonyl fluoride reacted with 4-hydroxybenzyl alcohol, followed with NBS to give the intermediate 4-(hexafluoropropene trimer-oxy)benzyl bromide or 4-(perfluorohexanesulfonyloxy) benzyl bromide. The intermediate was subjected to react with imidazole and then 1,3-propanesultone)	Carbon steel	1.5×10^{-4} M	^{1}H NMR; ^{19}F NMR; LC–MS; Surface tension measurement; PDP; EIS; SEM; XPS; QM	95.7	25	Mixed type of inhibitor	Chemical	Langmuir	329
29	Dodecyl 41-hydroxy-3,6,9,12,15,18,21,24,27,30,33,36,39-tridecaoxahentetracontyl phthalate	Carbon steel	1.0 × 10^{-2} M	FTIR; MS; ^{1}H NMR; Surface tension measurement; PDP; EIS; WL; SEM	94.9	30	Mixed type of inhibitor	Physical–chemical	Langmuir	198
30	N,N`-((oxalylbis(oxy))bis(ethane-2,1-diyl))bis(N,N-	Carbon steel	5 × 10^{-3} M	FTIR; ^{1}H NMR; MS; WL; PDP; EIS; Surface	94.84	25	Mixed type of inhibitor	Physical–chemical	Langmuir	89

	dimethyldodecan-1-aminium bromide)			tension measurement						
31	Natural polymer xanthan gum + Sodium dodecyl sulfate (SDS)	Mild steel	1000 ppm + 5 ppm SDS	WL; PDP; EIS; SEM; QM; UV	90.38	30	Mixed type of inhibitor	Physical	Langmuir	330
32	1-dodecyl-5-methyl-1H-benzo[d][1,2,3]triazol-1-ium bromide	Carbon steel	1×10^{-3} M	^{1}H NMR; ^{13}C NMR; FTIR; Surface tension measurement; Conductivity method; WL; PDP; EIS	97.3	25	Mixed type of inhibitor	Physical–chemical	Langmuir	200
33	N,N-dimethyl-4-(((1-methyl-2-phenyl-2,3-dihydro-1H-pyrazol-4-yl)imino)methyl)-N-hexadecylbenzenaminium bromide	Carbon steel	5×10^{-3} M	PDP; EIS; WL	99.6	60	Mixed type of inhibitor	Chemical	Langmuir	331
34	Imidazolium Gemini surfactant [C14-4-C14im]Br2	A3 Carbon steel	1.0×10^{-5} M	EIS; PDP; WL; QM	around 96	65	Mixed type of inhibitor	Chemical	Langmuir	209
35	Hexadecyldimethylisopropylammonium hydroxide (HEDIAOH)	Carbon steel	1×10^{-2} M	FTIR; ^{1}H NMR; Surface tension measurement; Conductivity method; WL; EIS; SEM; Biodegradability test	96.8	30	Mixed type of inhibitor	Physical	Langmuir	201
36	Alginate surfactant metal complexes (AS–Cu)	Carbon steel	5×10^{-3} M	FTIR; ^{1}H NMR; UV; EIS; PDP; WL; EDX; SEM	96.27	25	Mixed type of inhibitor	Chemical	Langmuir	332
37	N-(2-(2-mercaptoacetoxy)ethyl)-N,N-dimethyl dodecan-1-aminium bromide	Carbon steel	1×10^{-4} M	FTIR; ^{1}H NMR; MS; PDP; EIS; WL; QM; SEM; Surface tension measurement; Conductivity method	95.86	30	Mixed type of inhibitor	Chemical	Langmuir	108
38	l-phenylalanine methyl ester hydrochloride + Sodium dodecyl sulfate (SDS)	Mild steel	400 ppm + 5 ppm	WL; PDP; EIS; QM; UV; SEM; EDX	88.8	60	Mixed type of inhibitor	Physical–chemical	Langmuir	333
39	4-(N,N,N-dimethyldodecylammonium bromide)benzylidene-4-methoxybenzene-2-yl-amine	Carbon steel	5×10^{-3} M	FTIR; MS; PDP; EIS; WL; SEM	97.7	80	Mixed type of inhibitor	Chemical	Langmuir	334
40	Bis(p-(N,N,N-tetradecyldimethylammonium bromide)benzylidene thiourea (14-S-14)	Carbon steel	5×10^{-3} M	FTIR; ^{1}H NMR; WL; PDP; EIS; Surface tension measurement; SEM; Conductivity method	97.75	25	Mixed type of inhibitor	Physical–chemical	Langmuir	212

Table S5. Plant extracts as corrosion inhibitors for carbon steel in 1.0 M HCl media, the techniques, methods and instruments used for evaluation of the inhibition performance, and the results obtained.

	Inhibitor name	Sample	Optimum concentration	Techniques/ Investigations	Max Efficiency (%)	T (°C)	Electrochemical Type	Sorption	Isotherm model	[illegible]
1	Tunbergia fragrans	Mild steel	500 ppm	WL; PDP; EIS; SEM; EDS	81.1	25	Mixed type of inhibitor	Physical	Langmuir	[illegible]
2	Lavandula mairei extract	Mild steel	0.4 g·L^{-1}	WL; PDP; EIS; EDX; XPS; UV; DFT; MD	92	30	Mixed type of inhibitor	Chemical	Langmuir	[illegible]
3	Magnolia grandiflora leaves extract	Q235 steel	500 mg·L^{-1}	FTIR; PDP; EIS; SEM; AFM; QM; MD	88.2	25	Mixed type of inhibitor	Physical–chemical	Langmuir	[illegible]
4	Hyalomma tick extract	Carbon steel	3 g·L^{-1}	FTIR; PDP; EIS; SEM; EDS; AFM	95	25	Mixed type of inhibitor	Physical	Langmuir	[illegible]
5	Dardagan Fruit extract	Mild steel	3000 ppm	PDP; EIS; SEM; EDX; AFM; FTIR; UV; CA	97.5	25	Mixed type of inhibitor	-	Langmuir	[illegible]
6	Rosmarinus officinalis extract	XC 48 steel	400 ppm	WL; PDP; EIS; FTIR; LC–MS; MS; SEM; EDS; QM; MC	96.1	25	Mixed type of inhibitor	Physical	Langmuir	[illegible]
7	Papaver somniferum leaves/stems extract	Mild steel	600 ppm	SEM; EDX; AFM; UV; FTIR; XRD; CA; EIS; PDP; DFT; MC; MD	97.65	-	Mixed type of inhibitor	Physical–chemical	-	[illegible]
8	Aloysia citrodora leaves extract	Mild steel	600 ppm	FTIR; UV; EIS; PDP; SEM; AFM; CA; DFT; MC; MD	94	-	Mixed type of inhibitor	Physical–chemical	Langmuir	[illegible]
9	Ziziphora leaves extract	Mild steel	800 ppm	UV; FTIR; SEM; AFM; CA; OCP; EIS; PDP; MC; MD	93	-	Mixed type of inhibitor	Physical–chemical	Langmuir	[illegible]
10	Extract of tangerine peels (ETP) mediated copper nanoparticles (ETP-CuNPs)	X80 steel	1.0 g·L^{-1}	UV; FTIR; XRD; SEM; EDX; TEM; Antimicrobial assay; Biocorrosion monitoring; WL; EIS; PDP	95.3 ± 0.5	30	Mixed type of inhibitor	Physical	Langmuir	[illegible]
11	Pterocarpus santalinoides leaves extract	Carbon steel	0.7 g·L^{-1}	OCP; EIS; LPR; PDP; SEM; EDX; AFM; UV	94.02	60	Mixed type of inhibitor	Chemical	-	[illegible]
12	Laurus nobilis leaves extract	Carbon steel	400 ppm	UV; FTIR; SEM; AFM; EIS; PDP; CA; DFT; MC; MD	92	-	Mixed type of inhibitor	Chemical	Langmuir	[illegible]
13	Rosa canina fruit extract	Mild steel	800 ppm	UV; FTIR; EIS; PDP; SEM; EDS; CA; MD; QM	85.7	-	Mixed type of inhibitor	-	-	[illegible]

14	Chinese gooseberry fruit shell extract	Carbon steel	1000 ppm	OCP; EIS; PDP; SEM; AFM; CA; FTIR; UV; WL; DFT; MC; MD	94	25	Mixed type of inhibitor	Physical–chemical	Langmuir	[illegible]
15	Borage flower extract	Mild steel	800 ppm	FTIR; UV; WL; EIS; PDP; SEM; AFM; CA; QM; DFT; MC; MD	91	-	Mixed type of inhibitor	Physical	Langmuir	[illegible]
16	Ficus tikoua leaves extract	Carbon steel	1000 ppm	FTIR; EIS; PDP; SEM; DFT	95.80	25	Mixed type of inhibitor	Chemical	Langmuir	[illegible]
17	Invasive brown seaweed Sargassum muticum extract	Carbon steel	1 g·L^{-1}	WL; OCP; PDP; EIS; SEM; XPS	97	30	Mixed type of inhibitor	Chemical	Langmuir	[illegible]
18	Peganum harmala seed extract	Mild steel	800 ppm	FTIR; UV; OCP; PDP; EIS; SEM; AFM; CA; MC; DFT	95	-	Mixed type of inhibitor	Chemical	Freundlich	[illegible]
19	Eriobotrya japonica Lindl (EJL) extract	Mild steel	800 ppm	FTIR; UV; EIS; PDP; SEM; AFM; MC; MD; QM	92.8	-	Mixed type of inhibitor	Physical–chemical	Langmuir	[illegible]
20	Organic–inorganic hybrid complex based on Cissus quadrangularis (CQ) plant extract and zirconium acetate (CQ-ZrAc)	Mild steel	1100 ppm	GC–MS; FTIR; XRD; SEM; EDX; UV; WL; OCP; PDP; EIS; DFT	89.87	30	Mixed type of inhibitor	Physical	Langmuir	[illegible]
21	Pineapple stem extract	Mild steel	1000 ppm	WL; PDP; EIS; SEM; EDX; UV	97.6	65	Mixed type of inhibitor	Physical–chemical	Langmuir	[illegible]
22	Lemon Balm extract	Mild steel	800 ppm	FTIR; UV; Ram; OCP; EIS; PDP; AFM; SEM; QM; DFT; MC; MD	94.6	-	Mixed type of inhibitor	-	-	[illegible]
23	Mangifera indica (mango) leaves extract	Mild steel	1000 ppm	UV; FTIR; EIS; PDP; SEM; AFM; CA; MC; MD; RDF; QM; DFT	92	-	Mixed type of inhibitor	-	Langmuir	[illegible]
24	Primula vulgaris extract	Mild steel	1000 ppm	FTIR; UV; SEM; AFM; CA; OCP; PDP; EIS; MC; MD; DFT	95.50	-	Cathodic-type	-	Langmuir	[illegible]
25	Citrullus lanatus fruit (CLF) extract	Mild steel	800 ppm	UV; FTIR; OCP; EIS; PDP; SEM; AFM; CA; DFT; MC; MD	91	-	Mixed type of inhibitor	Physical–chemical	Langmuir	[illegible]
26	Mustard seed extract	Mild steel	200 mg·L^{-1}	FTIR; UV; OCP; PDP; EIS; SEM; AFM; WL; MC; MD; DFT	97	25	Mixed type of inhibitor	Physical–chemical	Langmuir	[illegible]
27	Sweet melon peel extract	Mild steel	0.5 g·L^{-1}	WL; PDP; OM	91.59	45	Anodic-type	Physical–chemical	Langmuir	[illegible]
28	Sunflower seed hull extract	Carbon steel	400 ppm	FTIR; GC–MS; PDP; EIS; UV	98.46	-	Mixed type of inhibitor	Physical	Langmuir	[illegible]

29	Ginkgo leaf extract	X70 steel	200 mg·L^{-1}	FTIR; EIS; PDP; OCP; PZC; SEM; AFM; QM	92.5	45	Mixed type of inhibitor	Physical–chemical	Langmuir	
30	Urtica dioica leaves extract	Mild steel	800 ppm	OCP; EIS; PDP; SEM; EDS; AFM; MC; MD; QM	92	-	Mixed type of inhibitor	Physical–chemical	Langmuir	[illegible]
31	Rollinia occidentalis extract	Carbon steel	1 g·L^{-1}	WL; EIS; PDP; UV; FTIR; SEM	85.7	25	Mixed type of inhibitor	Physical	Langmuir	[illegible]
32	Glycyrrhiza glabra leaves extract	Mild steel	800 ppm	OCP; EIS; PDP; AFM; CA; MD; MC; QM	88	-	Mixed type of inhibitor	Physical–chemical	Langmuir	[illegible]
33	Glycine max leaves extract	Mild steel	2 g·L^{-1}	PDP; EIS; SEM; EDS; DFT	94.07	25	Mixed type of inhibitor	Physical	Langmuir	[illegible]
34	Pisum sativum (green pea) peels extract	Mild steel	400 mg·L^{-1}	DFT; FTIR; UV; WL; PDP; EIS; SEM; AFM; IC	91	-	Mixed type of inhibitor	Physical	Langmuir	[illegible]
35	Pistachio shell extract	Mild steel	800 ppm	FTIR; UV; OCP; EIS; PDP; SEM; AFM; MC; MD; DFT	92.72	-	Mixed type of inhibitor	Physical–chemical	Langmuir	[illegible]
36	Musa paradisica (Banana) peels (raw) extract	Mild steel	300 mg·L^{-1}	WL; PDP; EIS; OCP; AFM; HPLC; UV; FTIR	90	26	Anodic-type	-	Langmuir	[illegible]
37	Thymus vulgaris extract	Mild steel	1000 ppm	FTIR; UV; Ram; OCP; PDP; EIS; SEM; CA; AFM; WL; MC; MD; DFT	95	25	Mixed type of inhibitor	Physical–chemical	Langmuir	[illegible]
38	Lilium brownii leaves extract	X70 steel	200 mg·L^{-1}	FTIR; OCP; PDP; EIS; SEM; AFM; DFT; MD	85.70	25	Mixed type of inhibitor	Physical–chemical	Langmuir	[illegible]
39	Lagerstroemia speciosa leaf extract	Mild steel	500 ppm	GC–MS; FTIR; WL; EIS; PDP; SEM; AFM; EDX; DFT; MD	94.41	60	Mixed type inhibition with anodic predominance	Physical–chemical	Langmuir	[illegible]
40	Alkaloids extract (AE) from Geissospermum laeve	C38 steel	100 mg·L^{-1}	EIS; PDP; SEM; CPC; HPLC; TLC; ^{1}H NMR; ^{13}C NMR	92	25	Mixed type inhibition with cathodic predominance	Physical	Langmuir	[illegible]
41	Banana leaves water extract	X70 steel	600 mg·L^{-1}	FTIR; OCP; PDP; EIS; SEM; AFM; DFT; MD	89.8	25	Mixed type of inhibitor	Physical–chemical	Langmuir	[illegible]
42	Phenolic extract of Ammi visnaga	Mild steel	0.15 g·L^{-1}	HPLC; PDP; EIS	93	-	Mixed type of inhibitor	Chemical	Temkin and El-Awady	[illegible]
43	Stachys byzantina (SB) leaves extract	Mild steel	1200 ppm	SEM; EDX; AFM; CA; FTIR; UV; XRD; Ram; EIS; PDP; MC; MD; DFT	97	-	Mixed type of inhibitor	Physical–chemical	Langmuir	[illegible]
44	Aqueous extract of Artemisia Herba Alba	Mild steel	0.4 g·L^{-1}	WL; EIS; PDP; SEM; EDX; XPS; DFT; MD	92	30	Mixed type of inhibitor	-	Langmuir	[illegible]

45	Methanolic extract of microalgae Chlorococcum sp. (MEC)	Mild steel	11.7 ppm	GC–MS; DFT; WL; EIS; PDP; SEM; OM; AFM	94.32	30	Mixed type of inhibitor	-	Temkin	[illegible]
46	Opuntia elatior fruit extract	Mild steel	500 ppm	FTIR; ^{1}H NMR; WL; PDP; EIS; SEM; XRD	≈88	30	Mixed type of inhibitor	Physical	Temkin	[illegible]
47	Heracleum persicum seeds phytoextract	Mild steel	400 ppm	FTIR; UV; SEM; AFM; CA; WL; PDP; EIS; Ram; DFT; MC; MD	96	25	Mixed type of inhibitor	Physical–chemical	Langmuir	[illegible]
48	Castor oil-based corrosion inhibitor	Mild steel	140 µM	OCP; EIS; PDP; AFM; SEM; EDX; DFT	92.8	25	Mixed type of inhibitor	Chemical	Langmuir	[illegible]
49	Anisole derivatives	Mild steel	1×10^{-3} M	OCP; PDP; EIS; SEM; DFT; MD	86.8	-	Mixed type of inhibitor	Chemical	Langmuir	[illegible]
50	Natural nutmeg oil	Carbon steel	500 ppm	WL; PDP;PDAP; DFT; MC	94.73	-	Mixed type of inhibitor	Physical–chemical	Langmuir	[illegible]
51	Phellodendron chinense Schneid	Carbon steel	4 g·L^{-1}	FTIR; LC–MS; EIS; PDP; WL; SEM; DFT; MD	92.5	-	Mixed type of inhibitor	-	-	[illegible]

Table S6. Polymers as corrosion inhibitors for carbon steel in 1.0 M HCl media, the techniques, methods and instruments used for evaluation of the inhibition performance, and the results obtained.

	Inhibitor name	Sample	Optimum concentration	Techniques/ Investigations	Max Efficiency (%)	T (°C)	Electrochemical Type	Sorption	Isotherm model	Ref
1	8-Hydroxyquinoline based chitosan derived carbohydrate polymer	Mild steel	$1{\times}10^{-2}$ g/L	FTIR; ^{1}H NMR; ^{13}C NMR; EIS; PDP; SEM; EDS; UV; DFT; MC	93.9	-	Mixed type of inhibitor	Chemical	Langmuir	374
2	Poly(butylene succinate)	Mild steel	600 ppm	FTIR; XRD; SEM; EDX; AFM; WL; PDP; EIS	78	25	Mixed type of inhibitor	Physical	Langmuir	375
3	Poly(o-toluidine) with zinc or lanthanum additives	Carbon steel	100 ppm + 5 mM ($ZnCl_2$/$LaCl_3$)	FTIR; XRD; TEM; SEM; EDX; UV; OCP; PDP; EIS; DFT; MD	96.8 ($ZnCl_2$)/98.9($LaCl_3$)	30	Mixed type of inhibitor	Physical–chemical	Langmuir	376
4	P(o-phenylenediamine)	Mild steel	15 ppm	WL; PDP	95	25	Mixed type of inhibitor	Physical	Langmuir	377
5	Hyperbranched poly(cyanurateamine)	Mild steel	2 mg·L^{-1}	FTIR; ^{1}H NMR; ^{13}C NMR; WL; PDP; EIS; SEM; AFM	98	25	Mixed type of inhibitor	Physical	Langmuir	231
6	Poly (naphthylamine-formaldehyde)	Mild steel	100 mg·L^{-1}	WL; PDP; EIS	99.90	30	Mixed type of inhibitor	Physical	Langmuir	378
7	Poly(aniline-formaldehyde)	Mild steel	10 ppm	WL; PDP; EIS; AFM	94.52	-	Mixed type of inhibitor	-	Langmuir	230
8	Poly(4-Vinylpyridine) and Potassium Iodide	Mild steel	100 mg·L^{-1} + 0.1% KI	WL; PDP; EIS	97.39	25	Mixed type of inhibitor	Physical–chemical	Langmuir	233

9	Poly(ortho-ethoxyaniline) (Mw = 13,000 g·mol^{-1})	Mild steel	100 ppm	GPC; UV; FTIR; EIS; PDP; LPR	98	-	-	Chemical	Temkin	379
10	P(2-aminobenzothiazole)	Mild steel	12.7 × 10^{-6}	CV; WL; PDP; EIS; SEM	73.12	25	Anodic inhibitor	Physical	Temkin	380
11	Poly(vinyl alcohol-cysteine)	Mild steel	0.6 wt%	UV; FTIR; SEM; EDX; Conductivity method; PDP; EIS; WL	95.39	-	Mixed type of inhibitor	Physical	EL-Awady	234
12	Poly (vinyl alcohol-o-methoxy aniline)	Mild steel	2000 ppm	WL; PDP; EIS; SEM	97.21		Mixed type of inhibitor	Physical–chemical	Langmuir	238
13	Poly(vinyl alcohol–proline)	Mild steel	0.6 wt%	FTIR; XRD; SEM; EDX; Conductivity method; PDP; EIS	94.82	30	Mixed type of inhibitor	Physical	Langmuir	381
14	Polyvinyl-Alcohol	Carbon steel	200 ppm	WL; PDP; EIS	93	25	Mixed type of inhibitor	Physical–chemical	Langmuir	382
15	Polyvinyl alcohol–sulfanilic acid water soluble composite	Mild steel	6000 ppm	FTIR; WL; PDP; EIS	95.2	-	Mixed type of inhibitor	Physical	Langmuir	383
16	Poly (vinyl alcohol – aniline) water soluble composite	Mild steel	2000 ppm	WL; PDP; EIS	92	-	Mixed type of inhibitor	-	Langmuir and Temkin	384
17	Poly-levodopa	Q235 Carbon steel	300 mg·L^{-1}	FTIR; UV; PDP; EIS; SEM; CL-SM	97.81	-	Mixed type of inhibitor	Physical	Langmuir	385
18	Modified polyaspartic acid	Carbon steel	80 ppm	FTIR; ^{1}H NMR; OCP; PDP; EIS; WL; SEM; AFM; DFT	90.39	30	Mixed type of inhibitor	Physical–chemical	Langmuir	386
19	Photo-cross-linked poly((E)-(1-(5-(4-(3-(4-chlorophenyl)-3-oxoprop-1-enyl)phenoxy)pentyl)-1H-1,2,3-triazol-4-yl)methyl acrylate)	Mild steel	15 ppm	FTIR; ^{1}H NMR; UV; EIS; PDP; SEM	99.1	-	Mixed type of inhibitor	Physical–chemical	Langmuir	235
20	Poly(4-vinylpyridine-hexadecyl bromide)	Mild steel	300 mg·L^{-1}	^{1}H NMR; WL; PDP; EIS	95	-	Cathodic inhibitor	Chemical	Langmuir	387
21	Poly (acrylamide-vinyl acetate)	Carbon steel	500 ppm	WL; PDP; EIS; SEM; QM; MEP	97.17	30	Mixed type of inhibitor	Physical–chemical	Langmuir	232
22	Terpolymers (hydrochloride salt of N,N-diallylmethionine 3. Cationic monomers 2 and 3 underwent alternate copolymerization with SO_2 in dimethyl sulfoxide)	Mild steel	70.2 µM	FTIR; ^{1}H NMR; TGA; OCP; EIS; PDP; XPS; SEM; EDX	99.3	60	Anodic inhibitor	Physical–chemical	Langmuir, Temkin, Freundlich	388
23	Sodium carboxymethyl cellulose (Na-CMC)	Mild steel	0.04 wt%	WL; EIS; PDP; SEM	78	25	Mixed type of inhibitor	Physical–chemical	Langmuir	239
24	N, N, 1-tri(oxiran-2-ylmethoxy)-5-((oxiran-2-ylmethoxy)thio)-1H-1,2,4-triazol-3-amine	C38 carbon steel	1000 ppm	DFT; MC; MD; ^{1}H NMR; ^{13}C NMR; PDP; EIS; SEM	92.4	-	Mixed type of inhibitor	Physical–chemical	Langmuir	229

Table S7. Polymeric nanoparticles as corrosion inhibitors for carbon steel in 1.0 M HCl media, the techniques, methods and instruments used for evaluation of the inhibition performance, and the results obtained.

	Inhibitor name	Sample	Optimum concentration	Techniques/ Investigations	Max Efficiency (%)	T (°C)	Electrochemical Type	Sorption	Isotherm model	Ref
1	Poly 12-(3-amino phenoxy) dodecane-1-thiol surfactant self assembled on silver nanoparticles	Carbon steel	375 ppm	FTIR; ^{1}H NMR; UV; XRD; TGA; Surface tension measurements; TEM; WL; PDP; EDX	87.59	25	Mixed type of inhibitor	-	-	246
2	Elaeis guineensis (EG) and silver nanoparticles (AgNPs)	Mild steel	10% v/v	WL; EIS; PDP; XRD; SEM; AFM; TEM; EDX	94.1	-	Mixed type of inhibitor	Physical	Langmuir	389
3	Onion mesocarp-nickel nanocomposites	X80 steel	20% v/v	UV; TEM; DLS; XRD; SEM; EDS; FTIR; WL; EIS; OCP; PDP; EFM; LPR; AFM	97.8	50	Mixed type of inhibitor	Physical	-	390
4	Organic nanoparticles of acetohydrazides (N′((E)-benzylidene)-2-(4-((E) hydrazonomethyl)phenoxyl)acetohydrazide nanoparticles)	Mild steel	1×10^{-3} M	^{1}H NMR; ^{13}C NMR; FTIR; TEM; XRD; DLS; PDP; EIS; SEM; AFM	96.7	25	Mixed type of inhibitor	Physical	Langmuir	391
5	Polyvinylpyrrolidone stabilized crystalline super-paramagnetic nanoparticles	Mild steel	0.10 wt%	TEM; XRD; PDP; EIS; DLS; Zeta potential analysis	98.39	30	Mixed type of inhibitor	-	-	392
6	Fe_3O_4@SiO_2 modified with hyperbranched polyglycerol	Mild steel	300 ppm	FTIR; XRD; TEM; TGA; AFM; OCP; LPR; EIS; WL; DFT; MC; MD	89.92	25	Mixed type of inhibitor	Physical–chemical	Langmuir	393
7	Ternary glycine-functionalized graphene/Fe_3O_4 nanocomposite	Mild steel	50 ppm	XRD; FTIR; EDX; SEM; TEM; WL; PDP; EIS	98.18	60	Cathodic type	Chemical	Langmuir	394
8	Polydopamine (PDA) nanoparticles	Mild steel	5 mg·L^{-1}	FTIR; UV; Zeta potential analysis; SEM; EDS; AFM; CA; XRD; OCP; PDP; EIS	99	-	Mixed type of inhibitor	-	-	242
9	Electrospun TiO2-nanofibers/Schiff base phenylalanine composite	Mild steel	300 mg·L^{-1}	FTIR; SEM; EDX; TEM; XPS; TGA; XRD; OCP; PDP; LPR EIS;	97.9	30	Mixed type of inhibitor	Physical–chemical	Langmuir	247
10	Nonionic amphiphilic chitosan nanoparticles	Carbon steel	250 ppm	^{1}H NMR; FTIR; DLS; PDP; EIS; CA; SEM	97.3	-	Mixed type of inhibitor	-	-	241
11	Core–shell preparing poly(2-acrylamido-2-methylpropane sulfonic acid) (PAMPS)-Na magnetite nanogel	Carbon steel	250 ppm	FTIR; TEM; PDP; EIS; SEM	97.21	45	Mixed type of inhibitor	Chemical	Langmuir	395

12	Chitosan grafted with poly(ethylene glycol) silver nanoparticles	Carbon steel	1×10^{-3} M	FTIR; ^{1}H NMR; XRD; TEM; EDX; PDP; EIS	92.75	25	Mixed type of inhibitor	-	-	396
13	Graphene oxide-chitosan-ZnO hybrids	Mild steel	500 ppm	FTIR; UV; SEM; AFM; TEM; PDP; EIS; WL	85.61	25	Mixed type of inhibitor	Physical–chemical	Langmuir	397
14	Chitosan-cobalt nanocomposites	Mild steel	300 mg·L^{-1}	UV; FTIR; TEM; EDAX; SEM; PDP; EIS; AFM	97	-	Mixed type of inhibitor	Physical–chemical	Frumkin	398
15	Cysteine based silver-gold nanocomposite (Cys/Ag-Au NCz)	Mild steel	300 ppm	OCP; FTIR; XRD; UV; SEM; EDX; TEM; TGA; WL; PDP; EIS	96.01	30	Mixed type of inhibitor	Physical	Langmuir	399
16	Tangerine peels extract mediated silver nanoparticles	X80 steel	1.0 g·L^{-1}	UV, XRD, TEM, EDX, DLS, FTIR, WL, OCP, EIS, PDP, SEM	93.9	30	Mixed type of inhibitor	Physical	Langmuir	400
17	Cobalt ferrite nanoparticles dispersed in silica matrix	Mild steel	1.2 g·L^{-1}	FTIR; ^{1}H NMR; UV; XRD; TGA; Surface tension measurements; TEM; WL; PDP; EDX	≈94	60	-	Physical–chemical	Langmuir	401
18	Magnetite (Fe_3O_4) polymer composite (Fe_3O_4/AM-co-AA-Na composite)	Carbon steel	100 ppm	FTIR; UV; TEM; PDP; EIS	99.75	-	-	-	-	402
19	Spherical polydopamine (PDA) nanoparticles	Mild steel	40 mg/L	FTIR; SEM; DLS; WL; PDP; EIS; AFM	86.42	-	Mixed type of inhibitor	Physical–chemical	Langmuir	403
20	Silver AMPS/NIPAm hybrid polymer (2-acrylamido-2-methylpropane sulfonic acid (AMPS)/N-isopropylacrylamide (NIPAm)-Ag nanogel) (AMPS/NIPAm-Ag NPs)	Carbon steel	250 ppm	UV; TEM; XRD; DLS; FTIR; EIS; PDP	81.46	-	Mixed type of inhibitor	-	-	404
21	Magnetite (Fe_3O_4) nanoparticles coated with rosin amidoxime	Mild steel	150 ppm	FTIR; TEM; DLS; PDP; EIS	96.8	-	Mixed type of inhibitor	-	-	405
22	Zinc oxide nanoparticles	Carbon steel	0.02 g/L	XRD; SEM; PDP; EIS	92.25	30	Mixed type (predominantly cathodic type) of inhibitor	Physical–chemical	Langmuir	406
23	Self-stabilized magnetic polymeric composite nanoparticles of coated poly-(sodium 2-acrylamido-2-methylpropane sulfonate-co-styrene)/magnetite (PAMPS-Na-co-St/Fe_3O_4)	Carbon steel	250 ppm	FTIR; TEM; TGA; Surface tension; PDP; EIS	99.7	-	Mixed type of inhibitor	-	-	407
24	N-isopropyl acrylamide co-polymer nanogel	Carbon steel	250 ppm	FTIR; TEM; DLS; PDP; EIS	94.8	-	Mixed type of inhibitor	-	-	408
25	Aspartic di-dodecyl ester hydrochloride acid (ADH) and	Carbon steel	1000 ppm	FTIR; PDP; QM; SEM; EDX;	91.5	30	Mixed type of inhibitor	Physical–chemical	Langmuir	409

its ZnO-NPs derivative (ADH-ZnO)	
